# Arterial Stiffness in Patients With Renal Transplantation; Associations With Co-morbid Conditions, Evolution, and Prognostic Importance for Cardiovascular and Renal Outcomes

**DOI:** 10.3389/fcvm.2019.00067

**Published:** 2019-05-24

**Authors:** Maria Korogiannou, Efstathios Xagas, Smaragdi Marinaki, Pantelis Sarafidis, John N. Boletis

**Affiliations:** ^1^Department of Nephrology and Renal Transplantation Unit, Laiko General Hospital, Medical School, National and Kapodistrian University of Athens, Athens, Greece; ^2^Department of Nephrology, Hippokration Hospital, Aristotle University of Thessaloniki, Thessaloniki, Greece

**Keywords:** arterial stiffness, transplantation, chronic kidney disease, augmentation index, cardiovascular events

## Abstract

Patients with chronic kidney disease (CKD), particularly those with end-stage renal disease (ESRD), are at increased risk of cardiovascular events and mortality. The spectrum of arterial remodeling in CKD and ESRD includes atheromatosis of middle-sized conduit arteries and, most importantly, the process of arteriosclerosis, characterized by increased arterial stiffness of aorta and the large arteries. Longitudinal studies showed that arterial stiffness and abnormal wave reflections are independent cardiovascular risk factors in several populations, including patients with CKD and ESRD. Kidney transplantation is the treatment of choice for patients with ESRD, associated with improved survival and better quality of life in relation to hemodialysis or peritoneal dialysis. However, cardiovascular mortality in transplanted patients remains much higher than that in general population, a finding that is at least partly attributed to adverse lesions in the vascular tree of these patients, generated during the progression of CKD, which do not fully reverse after renal transplantation. This article attempts to provide an overview of the field of arterial stiffness in renal transplantation, discussing in detail available studies on the degree and the associations of arterial stiffness with other co-morbidities in renal transplant recipients, the prognostic significance of arterial stiffness for cardiovascular events, renal events and mortality in these individuals, as well as studies examining the changes in arterial stiffness following renal transplantation.

## Introduction

Patients with chronic kidney disease (CKD) and particularly patients with end-stage renal disease (ESRD) are individuals with an early and marked increase in arterial stiffness, characterized by alterations in the viscoelastic properties of large arteries (1–3). Although the mechanisms for the development of increased arterial stiffness in CKD are complex and not yet fully clarified, data from experimental and clinical studies have shown that both classical and non-classical cardiovascular risk factors, i.e., factors related to CKD, play an important role in arterial remodeling. Classical risk factors include age, hypertension, diabetes mellitus dyslipidemia, obesity, smoking, and others. Non-classical risk factors relate to a number of alterations relevant to CKD progression and the uremic milieu, such as vascular calcification generated by the disturbed metabolism of calcium and phosphate, excess stimulation of renin-angiotensin-aldosterone system (RAAS), endothelial dysfunction and chronic inflammation present in advanced CKD and others, that can play an important role in adverse arterial remodeling (4–6).

Several epidemiological studies have shown that patients with reduced renal function are at increased risk for cardiovascular events and all-cause mortality (7). The association between CKD and cardiovascular events is present even in patients with mild decrease in renal function which has not yet caused a noticeable increase in serum creatinine (8). As described above, traditional cardiovascular risk factors are highly prevalent in patients with CKD and greatly confer to the risk of developing cardiovascular disease. However, even when such factors are taken into account for risk prediction, they fail to accurately predict survival, and residual risk remains, possibly associated with specific alterations taking place in CKD. Inclusion of alterations such as arterial stiffness, may help better assess cardiovascular risk in these patients (9). Prospective studies in different populations, including several in ESRD patients, showed that parameters reflecting arterial stiffness, such as PWV, or the adverse morphology of the pulse wave, such as increased augmentation pressure and augmentation index are strong and independent indicators of cardiovascular and total mortality (2, 10–12). Meta-analyses of such studies including large number of patients had similar results (13–15). At the same time, several studies have shown that blood pressure (BP) at the central aorta are generally more closely associated to the incidence of cardiovascular events and total mortality in several populations, including patients with CKD and ESRD, compared to peripheral BP recorded at the level of brachial artery (11, 14–16), indicating that central BP is a better marker of cardiovascular risk.

Kidney transplantation is the treatment of choice in patients with ESRD as it is associated with at least 2-fold longer survival in relation to hemodialysis or peritoneal dialysis and significant benefits in the quality of life of patients. However, the risk of cardiovascular death in transplanted patients remains significantly higher than that in general population (17, 18). It was suggested that part of the high cardiovascular risk of these patients is attributed to irreversible lesions in the vascular tree, which are created during the period prior to renal transplant and which do not fully reverse after it. Herein we discuss in detail available studies on arterial stiffness in patients with renal transplantation, that examined the levels of arterial stiffness and its with co-morbidities and graft function, the prognostic significance of arterial stiffness for cardiovascular events, renal events, and mortality in this population, as well as the natural course of arterial stiffness following renal transplantation, aiming to provide an overview of this important field.

## Arterial Stiffness and Central (Aortic) BP; Physiology, Pathophysiology, and Assessment

Arterial stiffness is a term describing the loss of flexibility of elastic arteries, i.e., the aorta and the other major arteries. Elastic arteries (or conducting arteries) receive blood directly from the heart and are the largest arteries of the body, those closest to the heart. The walls of these arteries have abundant elastic fibers, which, apart from the solidity, offer the ability of dilatation of the vessels; thus aorta and major arteries can through dilatation to practically “store” part of the blood volume during heart systole and forward it to the periphery during diastole. This cushioning function is a fundamental part of the circulation as it helps to avoid major changes in BP and keep the flow of blood as smooth as possible in the time between two heart beats (5, 19–21). Arterial stiffness is the result of structural and functional changes in the arterial tree and increases progressively with age regardless of the levels of the BP (22). Thus, arterial stiffness is a complex result of the effect of aging and that of classic and non-classic cardiovascular risk factors on the arterial tree.

**Figure 1:**
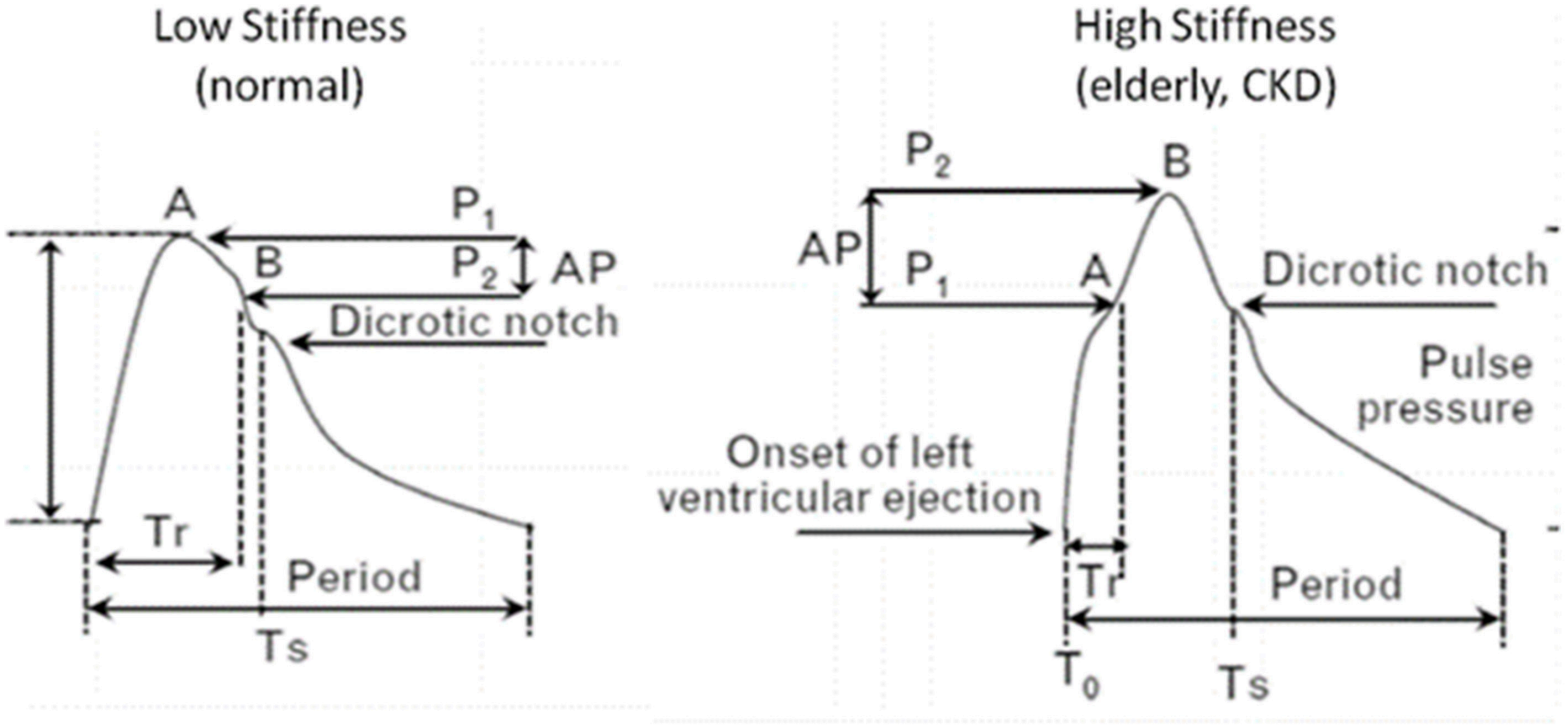
Pressure waveforms and characteristics in patients with high and low arterial stiffness. In increased arterial stiffness, increased velocity of the pulse wave results in earlier return of the wave to the aorta, i.e., during systole instead of diastole. Thus, it is added and increases SBP (augmentation pressure), while DBP is decreased. CKD, chronic kidney disease; Tr, arrival time of reflected waves at central aorta from the onset of left ventricular ejection (T0) to inflection point A; AP = P1–P2 the augmentation of aortic systolic pressure induced by the return of the reflected wave, where P1 is the pressure at the first inflection point A and P2 is the pressure at the second inflection point B; Augmentation index (AI) (%) is defined by the formula: AI = 100 × AP/PP, where PP is the aortic pulse pressure (systolic minus diastolic pressure); Ts, period from start to the end of systole (ejection duration).

In cases of increased arterial stiffness, the normal functioning of the aorta and large vessels is disrupted resulting major changes in the circulatory system (Figure 1). Normally, the initial arterial wave is produced by the left ventricle during contraction and travels to the periphery through a low resistance route, which keeps the mean blood pressure almost unchanged. When the pulse wave reaches the circumference, it is reflected and returns to the aorta. The points on which the reflex of the pulse wave occurs are the arterial intersections and the arterioles. In situations of increased arterial stiffness, due to the increased velocity of the pulse wave there is a faster return of reflecting arterial waves. These results in earlier return of the wave to the aorta, i.e., instead of returning to the aorta during the phase of dilation, as is normally the case, it returns during the contraction phase and therefore it is added and increases the systolic BP (SBP) by a BP degree, called “augmentation pressure.” Further to that the absence of the returning during diastole, results in DBP reduction. Thus, the increase in arterial stiffness, is associated with changes in BP, such as increased SBP, reduced diastolic blood pressure (DBP) and therefore increased pulse pressure (PP) (19, 22, 23, 23–25).

This increase in arterial stiffness is considered to be the main mechanism for the creation of isolated systolic hypertension, left ventricular hypertrophy, and heart failure with decreased cardiac output (26). Typically, increased arterial stiffness is an important factor in non-response of SBP in antihypertensive therapy which is a main characteristic of patients with resistant hypertension (27, 28). Furthermore, arterial stiffness has been shown to be a strong and independent cardiovascular risk factor in the general population, but also in patients with diabetes mellitus, hypertension, dyslipidemia, coronary artery disease, heart failure and, typically CKD and ESRD (2, 4, 10, 11, 15, 29, 30).

The velocity of transmission of the pulse wave along the arterial wall increases as we move from central to peripheral parts of the arterial tree, due to the change in the properties of the arterial wall (progressive change from “elastic type” to “muscular type” arteries). Due to the different distance from the reflection points, the morphology of the final pulse wave (i.e., the synthesis of promoted and reflected wave) at each point of the arterial tree is different from the others. Thus, the maximum SBP and therefore PP are different along the arterial tree. In normal conditions, the SBP at the level of the brachial artery is higher than at the level of the central arteries, while the diastolic and mean BP differ much less between these points. Especially in healthy young people, central (aortic) systolic BP is lower than peripheral up to 30 mmHg or more (31). This phenomenon of SBP and PP increase when moving from central to peripheral arteries is defined as “pulse pressure amplification.” This difference between peripheral and central BP is not always stable. The difference between central SBP or PP depends on many physiological factors (e.g., heart rate, geometry, and mechanical properties of the arterial tree, sex), but also on pathological factors (e.g., metabolic, inflammatory), and on the use of drugs (32). Typically, with increasing age and the relevant increase of the arterial stiffness in the aorta and the other elastic arteries, the difference between peripheral and central SBP and PP decreases significantly.

The heart, the kidneys and the large vessels that feed the brain are anatomically closer and more strongly influenced by the effects of central rather than brachial BP. Therefore, it is reasonable to assume that central BP relates more closely to target-organ damage and that it has an important predictive value as far as cardiovascular mortality concerned. A plethora of data suggests that central BP is better correlated with target organ damage, cardiovascular risk and events, and mortality than peripheral BP (16, 30, 33). Indeed, it appears that central BP is more closely related to the thickening of tunica intima and tunica media of the carotids as well as to the hypertrophy of the left ventricle of the heart compared to brachial BP (33–35). In addition, the reversion of left ventricular hypertrophy and of intima-media thickness of the carotid was associated with the change of central and not brachial BP (36, 37). Moreover, in subjects without established cardiovascular disease, central BP was superior to brachial BP in the prognosis of future cardiovascular events (33). In another study including patients with ESRD, only central BP and the reduction of augmentation pressure were independent predictive factors of total (and cardiovascular) mortality (30). A metanalysis that explored the predictive value of central pressures in the incidence of cardiovascular events and mortality, highlighted the independent and stronger predictive value of central BP over peripheral BP (15).

There are currently several methods available to determine arterial stiffness and central pressures in a non-invasive way. In clinical practice, pulse wave velocity (PWV) along the aorta is the main parameter used to determine arterial stiffness. PWV is defined as the speed of transmission of the pulse wave along the arterial wall and is calculated by the ratio of the distance between two points of the arterial tree to the time of transmission of the pulse wave between these two points (19), i.e.,

$$\begin{array}{l} \text{PWV}=\frac{\text{distance of two points of the arterial tree }\left(\text{in meters}\right)\;}{\text{ Time of transmission of the pulse wave between these points }\left(\text{in seconds}\right)} \end{array}$$

With the modern ways of PWV determination, the speed at which the pulse wave travels between two superficial points of the arterial tree can be calculated. Tonometric techniques (with devices such as Sphygmocor), ultrasonographic techniques, plethysmography, and indirect identification techniques (with devices such as Mobilograph) are used to determine PWV (11, 38–40). For example, with the tonometric method (which is the one used in most studies of the current field) the PWV over different segments of the arterial tree (most commonly carotid-femoral, carotid-radial or radial-femoral PWV) can be measured with simultaneous recording with two probes, or with two recordings referenced to a concurrently recorded ECG, with pulse wave transit time between the subsequent recording sites calculated with special software (11, 39, 40). With the same technique, by measuring at the level of a superficial accessible artery the pulse wave in the aorta can be determined and the augmentation pressure, augmentation index (AIx) and other parameters can be calculated. Initially, the waveform of the pulse wave is determined in a superficial artery (e.g., radial) and then, with the help of a mathematical function (generalized transfer function), the waveform in the aorta is estimated (40, 41). The waveform of the aortic pulse wave is analyzed in order to calculate the amplification pressure, the AIx and also the aortic (central) systolic and diastolic blood pressure, the duration of the ejection phase of the left ventricle and the time at which the reflecting wave appears. In a similar manner, the oscillometric devices record BP at the diastolic phase for ~10 s, build the brachial pulse waveforms, and then generate the aortic pulse waveform with generalized transfer function (40, 42, 43).

## Arterial Stiffness in Renal Transplant Recipients

### Cross-Sectional Studies Assessing the Levels of PWV and Its Associations With Co-existing Risk Factors and Co-morbidities

In previous years several cross-sectional studies aimed to assess the degree of arterial stiffness and explore its association to cardiovascular events and renal graft outcome in renal transplant recipients (Table 1). Bahous et al. conducted a study (44), at which aortic PWV was measured non-invasively in 101 living kidney donors and their 101 corresponding recipients and was compared to healthy volunteers (divided into 2 groups: one recipient-related through familial links and the other non-recipient related). Aortic PWV was significantly higher in donors and recipients than in healthy volunteers, even after adjustment for age, gender, and MAP (9.5 ± 2.5 m/s in donors vs. 12.0 ± 2.0 m/s in recipients vs. 8.5 ± 1.5 m/s in non-recipient related healthy volunteers vs. 8.9 ± 1.5 m/s in recipient-related healthy volunteers, with all comparisons between groups being statistically significant, *p* ≤ 0.01). The factors related to donor aortic PWV, evaluated at end of follow-up, were donor age, MAP, plasma glucose, smoking, and time since nephrectomy. The factors related to recipient PWV were age, MAP, and smoking habit (as in donors), but also graft rejection. When examining recipients with chronic allograft nephropathy, plasma creatinine doubling was associated with 2 factors after adjusting for age: acute rejection (*p* = 0.004) and donor PWV (*p* = 0.03) (44).

**Table 1:** Cross-sectional studies assessing the level of arterial stiffness and its associations with co-existing risk factors and co-morbidities and graft function.

**Study ID**	***N***	**Population characteristics**	**Follow-up/Time points**	**Arterial stiffness assessment**	**Results**
Bahous et al. (44)	202	Renal transplant recipients and their corresponding living kidney donors		aortic PWV *Complior*	Aortic PWV was significantly higher in donors and recipients than in healthy volunteers (9.5 ± 2.5 m/s in donors vs. 12.0 ± 2.0 m/s in recipients vs. 8.5 ± 1.5 m/s in non-recipient related healthy volunteers vs. 8.9 ± 1.5 m/s in recipient-related healthy volunteers, *p* ≤ 0.01). Factors related to donor aortic PWV: donor age, MAP, plasma glucose, smoking habits, and time since nephrectomy. Factors related to recipient PWV: age, MAP, smoking habits, graft rejection
Kolonko et al. (45)	142	Renal transplant recipients	Time point 8.4 ± 1.8 years after Tx	aortic PWV *Complior*	Traditional risk factors related to increased IMT and PWV: diabetes (IMT 0.67 ± 0.11 cm, PWV 14.5 ± 5.6 m/s, *p* < 0.01), LVH (IMT 0.67 ± 0.14 cm, PWV 13.5 ± 4.8 m/s, *p* < 0.001) and CVD (IMT 0.73 ± 0.13 cm, PWV 14.7 ± 5.6 m/s, *p* < 0.001). In multivariate regression analysis, PWV was explained by age (β 0.28, 95% CI: 0.125 to 0.435, *p* < 0.001) and pre-transplantation diabetes (β 0.242, 95% CI: 0.077 to 0.407, *p* < 0.01)
Kolonko et al. (46)	145	Renal transplant recipients	Time point 7.6 ± 2.7 years after	c–f PWV *SphygmoCor*,	Higher PWV (median 9.6/interquartile range: 3.9 vs. 8.0/3.3 m/s, *P* = 0.002) but borderline lower FMD (8.4% ± 5% vs. 9.9% ± 5.7%, *P* = 0.09) in patients that did not reach therapeutic BP goal as compared to those with good or borderline BP control. Increased LVH prevalence and higher PWV values along with the increase in number of antihypertensive drugs
Azancot et al. (47)	92+30 controls	Renal transplant recipients+age–matched CKD patients		c-f PWV *SphygmoCor*	No significant difference between transplant and CKD patients in IMT (0.768 ± 0.139 vs. 0.761 ± 0.126 mm, *P* = 0.134), PWV (7.98 ± 1.75 vs. 8.17 ± 1.84, *P* = 0.628), and ankle-brachial pressure index
Strózecki et al. (48)	104	Renal transplant recipients		c-f PWV *Complior*	Higher PWV in the CAC+ group than in patients without CAC (10.2 ± 2.2 vs. 8.6 ± 1.5 m/s respectively, *p* < 0.001) Sensitivity and specificity of PWV > 10.2 m/s as cut-off for detecting severe CAC (CS > 400) was 0.319 and 0.969, respectively, suggesting that PWV measurement could be useful in excluding severe CAC in RTRs
Pacek et al. (49)	17	Renal transplant recipients	Time point 3–7 days after Tx	24-h PWA *Schiller BR-102 Plus PWA system*	Significant correlation (*r* = 0.21, *P* < 0.05) between BMI and PWV. On multivariate linear regression analysis, with PWV as a dependent variable, the independent predictors were age, hemoglobin levels, and 24-h SBP (an age increase of 10 years was correlated with a 0.47 m/s increase in PWV, while a 1 g/dl hemoglobin increase correlated to a 0.933 m/s decrease in PWV and a 24-h SBP increase of 10 mm Hg was correlated with a PWV increase of 0.83 m/s)
Ayub et al. (50)	96	Renal transplant recipients		Aortic PWV *Transcutaneous Doppler flow recordings, foot-to-foot method*	Aortic PWV ranged from 4 to 14.2 m/s. Aortic PWV and e GFR (using the MDRD equation) were inversely correlated (Pearson correlation coefficient −0.427, *p* = 0.00), suggesting a probable predictive value of aortic PWV in graft outcomes
Czyzewski et al. (51)	83	Renal transplant recipients		aortic PWV *Complior*	Multivariable linear regression analysis, with PWV as a dependent variable, retained the following independent predictors in the final regression model: RDW (β 0.323, 95% CI 0.319–1.591, *p* = 0.004), age (β 0.297, 95% CI 0.023–0.106, *p* = 0.005), tacrolimus immunosuppression therapy (β −0.286, 95% CI −2.616 to −0.554, *p* = 0.004) and central DBP (β 0.185, 95% CI 0.004 to 0.122, *p* = 0.041)

Several years after the above observations, Kolonko et al. (45) reported on a cross-sectional study which included 142 stable renal transplant recipients at an average of 8.4 ± 1.8 years after transplantation in order to assess different markers of vascular injury (including PWV and IMT) and endothelial disfunction and explore their association with traditional and novel risk factors. A high prevalence of traditional cardiovascular risk factors was noted in the population studied. Left ventricular hypertrophy was present in 50% of the patients and atherosclerotic plaques were found in 31%. Mean IMT was 0.62 ± 0.13 mm and PWV 12.7 ± 4.4 m/s. Among the traditional risk factors, the only ones that were related to increased IMT and PWV were diabetes (IMT 0.67 ± 0.11 cm, PWV 14.5 ± 5.6 m/s, *p* < 0.01), LVH (IMT 0.67 ± 0.14 cm, PWV 13.5 ± 4.8 m/s, *p* < 0.001) and CVD (IMT 0.73 ± 0.13 cm, PWV 14.7 ± 5.6 m/s, *p* < 0.001). In multivariate regression analysis, PWV was associated with age (β 0.28, 95% CI: 0.125 to 0.435, *p* < 0.001) and presence of pre-transplantation diabetes (β 0.242, 95% CI: 0.077 to 0.407, *p* < 0.01) (45).

Another recently published interesting cross-sectional study by the same group (46), tried to explore the levels of arterial stiffness and endothelial dysfunction in association with the effectiveness of antihypertensive treatment in renal transplant recipients. The study included 145 renal transplant recipients 7.6 ± 2.7 years after transplantation on average and measurements of PWV with the Sphygmocor device, flow-mediated dilation (FMD) and nitroglycerin-mediated dilation (NMD) along with 24-h ambulatory BP monitoring were performed. Overall, there were only 29 patients (20%) with well-controlled BP and 33 (23%) with borderline BP control. Eighty three patients (57%) failed to achieve the target blood pressure despite antihypertensive treatment. The study revealed a significantly higher PWV (median 9.6/interquartile range: 3.9 vs. 8.0/3.3 m/s, *p* = 0.002) but borderline lower FMD (8.4% ± 5% vs. 9.9% ± 5.7%, *p* = 0.09) in patients that did not reach the therapeutic BP goal as compared to those with good or borderline BP control. Further analysis of patients in subgroups based on the number of antihypertensive drugs revealed a significant trend for increased LVH prevalence and higher PWV values with increased number of antihypertensive drugs (8.7 ± 2.9 m/s in the untreated group vs. 8.9 ± 2.0 m/s in patients treated with 1 drug vs. 9.5 ± 3.0 m/s in patients treated with 2 drugs vs. 9.4 ± 2.0 m/s in those treated with 3 drugs vs. 11.1 ± 3.5 m/s in patients treated with 4 drugs, *p* = 0.02). Interestingly, there was no significant difference in FMD, NMD, and IMT between these subgroups (46).

In contrast to the above, a study by Azancot et al. (47) aiming to evaluate risk factors associated with hypertension in renal transplant recipients and included 92 consecutive renal transplant recipients and 30 CKD patients with similar age, sex, renal function, and proteinuria found no significant difference between transplant and CKD patients in intima media thickness (IMT) (0.768 ± 0.139 vs. 0.761 ± 0.126 mm, *p* = 0.134), PWV (7.98 ± 1.75 vs. 8.17 ± 1.84, *P* = 0.628), and ankle-brachial pressure index, which was normal in 67 (72.8%) transplant and 20 (66.6%) CKD patients and abnormally high in 21 (22.8%) transplant and 8 (26.6%) CKD patients (47). In another cross-sectional analysis (48), Stróżecki et al. attempted to investigate relationship between coronary artery calcification (CAC) and PWV and assess the performance of PWV measurement in predicting CAC. One hundred and four consecutive renal transplant recipients were recruited to the study with a mean kidney transplant follow-up of 38 months. CAC measurement included determination of the mass of the calcifications, as well as the total calcium score (CS). Patients with CS > 0 were considered to have CAC and patients with CS > 400 were considered to have severe CAC. CAC was found in 72 patients (69%) (CAC+ group). Thirty three (32%) of study patients had CS > 400. Among CAC+ patients, median CS was 322.5 (range 2.1–3730.7). Renal transplant recipients without CAC were compared to the CAC+ group. PWV was higher in the CAC+ group than in patients without CAC (10.2 ± 2.2 vs. 8.6 ± 1.5 m/s, respectively, *p* < 0.001). In univariate analysis CS was significantly correlated with age, duration of hypertension, waist circumference, PWV, hemoglobin levels and serum glucose, whereas in multiple linear regression analysis CS was independently associated only with age and not with PWV. Sensitivity and specificity of PWV > 7.6 m/s as cut-off for detecting CAC >0 was 0.889 and 0.406, respectively. Sensitivity and specificity of PWV > 10.2 m/s for detecting severe CAC (CS > 400) was 0.319 and 0.969, respectively, suggesting that PWV measurement could be useful in excluding severe CAC in renal transplant recipients (48).

Finally, a small cross-sectional study (49) including 17 consecutive renal transplant recipients who underwent a 24-h ABPM and PWV measurement in the early post-operative period (3 to 7 days after transplantation) and to whom anthropometric measurements and laboratory parameters were obtained, tried to explore the association of the above-mentioned parameters with the risk of cardiovascular disease in these patients. There was a significant correlation (*r* = 0.21, *P* < 0.05) between overweight as defined by BMI and the PWV measurements. On multivariate linear regression analysis, with PWV as a dependent variable, the factors independently associated with it were age, hemoglobin levels and 24-h SBP. An age increase of 10 years was correlated with a 0.47 m/s increase in PWV, while a 1 g/dl hemoglobin increase correlated to a 0.933 m/s decrease in PWV. Finally, a 24-h SBP increase of 10 mm Hg was correlated with a PWV increase of 0.83 m/s (49).

### Cross-Sectional Studies on the Association of Arterial Stiffness and Graft Function

The association between arterial stiffness and graft function was investigated in 2 recently published cross-sectional studies (Table 1). In the first one (50), PWV measurement with ultrasound recordings was performed in 96 stable renal transplant recipients. The aortic PWV of the patients ranged from 4 to 14.2 m/s. The aortic PWV and the estimated GFR (using the MDRD equation) were inversely correlated (Pearson correlation coefficient was −0.427), and this correlation was statistically significant, suggesting a probable effect of arterial stiffness on graft outcomes (50). In the second study (51), PWV was measured in 83 renal transplant recipients, aiming to assess the degree and associations of arterial stiffness. Multivariable linear regression analysis, with PWV as a dependent variable, retained the following parameters as independent predictors of PWV in the final regression model: red blood cell distribution width (β 0.323, 95% CI 0.319 to 1.591, *p* = 0.004), age (β 0.297, 95% CI 0.023 to 0.106, *p* = 0.005), tacrolimus immunosuppression therapy (β −0.286, 95% CI −2.616 to −0.554, *p* = 0.004) and central DBP (β 0.185, 95% CI 0.004 to 0.122, *p* = 0.041) (51).

### Longitudinal Studies Assessing the Association of Arterial Stiffness With Cardiovascular Risk, Renal Outcomes, and Mortality in Renal Transplant Recipients

In addition to the aforementioned cross-sectional studies, few retrospective and prospective cohort studies have tried to explore the association of arterial stiffness indices with cardiovascular risk, renal outcomes and mortality in renal transplant recipients (Table 2).

**Table 2:** Longitudinal studies assessing the association of arterial stiffness with cardiovascular risk, renal outcomes, and mortality in renal transplant recipients.

**Study ID**	***N***	**Population characteristics**	**Follow-up/Time points**	**Arterial stiffness assessment**	**Results**
**RETROSPECTIVE STUDIES**					
Kim et al. (52)	171	171 ESRD pts eligible for Tx, in 84 of whom follow-up baPWV was available	Before Tx and 1 year after Tx	baPWV *VP-1000 BP203RPEII (Colin Company, Kyoto, Japan)*	Pre-transplant baPWV was higher in patients with history of CVD than in those without (18 ± 4.4 vs. 14.91 ± 2.65 m/s, *p* < 0.05) and was a strong predictor of CVD (OR1.003, 95% CI: 1.001 to 1.005, *p* < 0.05). The optimal cut-off value of baPWV for the detection of CVD was 15.91 m/s and this value was an independent predictor of CVD in RTRs (OR 6.3, *p* < 0.05). Moreover, the occurrence rate of CVD was significantly higher in patients with “high coronary calcium score” compared to those with a “low coronary calcium score” and baPWV was also significantly higher to the first when comparing the two groups (16.27 ± 3.93 vs. 14.79 ± 2.65 m/s, *p* < 0.05)
Dahle et al. (53)	1,040	Renal transplant recipients	Follow-up 4.2 years	Aortic PWV *Sphygmocor*	Each 1 m/s increase in PWV up to 12 m/s, was significantly associated with mortality (HR 1.36, 95% CI 1.14 to 1.62, *p* = 0.001). An interquartile range increase of 3.8 m/s tripled the risk of mortality (HR 3.21, 95% CI 1.63 to 6.31), an effect similar to the effect of 1 interquartile increase in age (21.6 years, with an estimated HR of 3.06, 95% CI 1.87 to 5.29)
Cheddani et al. (54)	220	Renal transplant recipients	Time points 3 months and 1 year after Tx Follow-up 5.5 years	c-f PWV *Complior*	c-f PWV 3 months after transplantation was an independent risk factor for mortality (HR: 1.38, 95% CI 1.18 to 1.62, *p* < 0.001). Mortality was also significantly associated with c-f PWV 12 months after transplantation (HR 1.34, 95% CI 1.1 to 1.64, *p* = 0.004), but surprisingly not with aortic stiffness change between 3 and 12 months (HR 1.09, 95% CI 0.71 to 1.76, *p* = 0.696)
**PROSPECTIVE STUDIES**					
Bahous et al. (55)	106	Renal transplant recipients	Follow-up 54.3 ± 28.9 months	aortic PWV *Complior*	Aortic PWV increased in RTRs independently of age and mean BP. Acute renal rejection (β 1.15, *p* = 0.01) and smoking (β 0.02, *p* = 0.025) were the principal factors modulating the increase of aortic PWV and the reduction of GFR. Occurrence of renal and/or cardiovascular events following transplantation was influenced by heart rate (HR, 7.16; *p* < 0.001) and PWV (HR. 0.25; *p* < 0.006)
Claes et al. (56)	253	Renal transplant recipients	Follow-up 3 years	c-f PWV *SphygmoCor*	When accounting for age, gender, and CV history, AC score (HR, 1.09 per 1 unit increase; 95% CI 1.02 to 1.17) and PWV (HR 1.45 per 1 m/s; 95% CI 1.16 to 1.8) remained an independent predictor of CV events in Cox-regression analyses. Using ROC the area under the curve for predicting CV events amounted to 0.80 and 0.72 for sum AC and PWV, respectively
Laucyte-Cibulskiene et al. (57)	37	Renal transplant recipients	Time points Before and year after Tx	c-f PWV, c-r PWV *SphygmoCor*	Pretransplant CRP level (HR 1.660, *p* = 0.007) and PWV ratio (cfPWV/crPWV) (HR 7.549, *p* = 0.045) predict cardiovascular events
Bahous et al. (58)	190	Renal transplant recipient-donor pairs	Follow-up 56 ± 18 months	Aortiv PWV *Complior*	Borderline significant association of donor aortic PWV with the composite outcome (occurrence of a fatal or nonfatal CV event and/or doubling of serum creatinine or development of ESRD) (RR 1.8, 95% CI 1 to 3.4, *p* = 0.05). When renal and CV outcomes were separately analyzed, recipient eGFR and donor PWV were significant determinants of the renal outcome (HR 0.26, 95% CI 0.14 to 0.4, *p* < 0.0001 and HR 1.9, 95% CI 1.2 to 3.0, *p* = 0.02, respectively) and previous history of CV events was the only significant determinant of the CV outcome (HR 3.5, 95% CI 2.1 to 8, *p* = 0.001).

#### Retrospective Studies

A cohort study (52) by Kim et al. included 171 ESRD patients eligible for kidney transplantation, in 84 of which follow-up brachial ankle PWV (baPWV) was available. The study aimed to assess the utility of arterial stiffness measurements as a marker in predicting cardiovascular disease in renal transplant recipients. The mean value of pre-transplant baPWV was 15.08 ± 3 m/s in ESRD patients and 93.4% had a higher baPWV value than healthy controls with same age and sex. Pre-transplant baPWV was higher in patients with a history of CVD than in those without CVD (18 ± 4.4 vs. 14.91 ± 2.65 m/s, *p* < 0.05) and was proved to be a strong predictor of CVD (OR 1.003, 95% CI: 1.001 to 1.005, *p* < 0.05). The optimal cut-off value of baPWV for the detection of CVD was 15.91 m/s with a sensitivity of 72.7% and specificity of 71.6% (area under curve 0.778, 95% CI 0.64 to 0.91, *p* < 0.05), and this value was an independent predictor of CVD in renal transplant recipients (OR 6.3, *p* < 0.05). Moreover, the occurrence rate of CVD was significantly higher in patients with “high coronary calcium score” compared to those with a “low coronary calcium score” and baPWV was also significantly higher in the first of the above two groups (16.27 ± 3.93 vs. 14.79 ± 2.65 m/s, *p* < 0.05).

**Figure 2:**
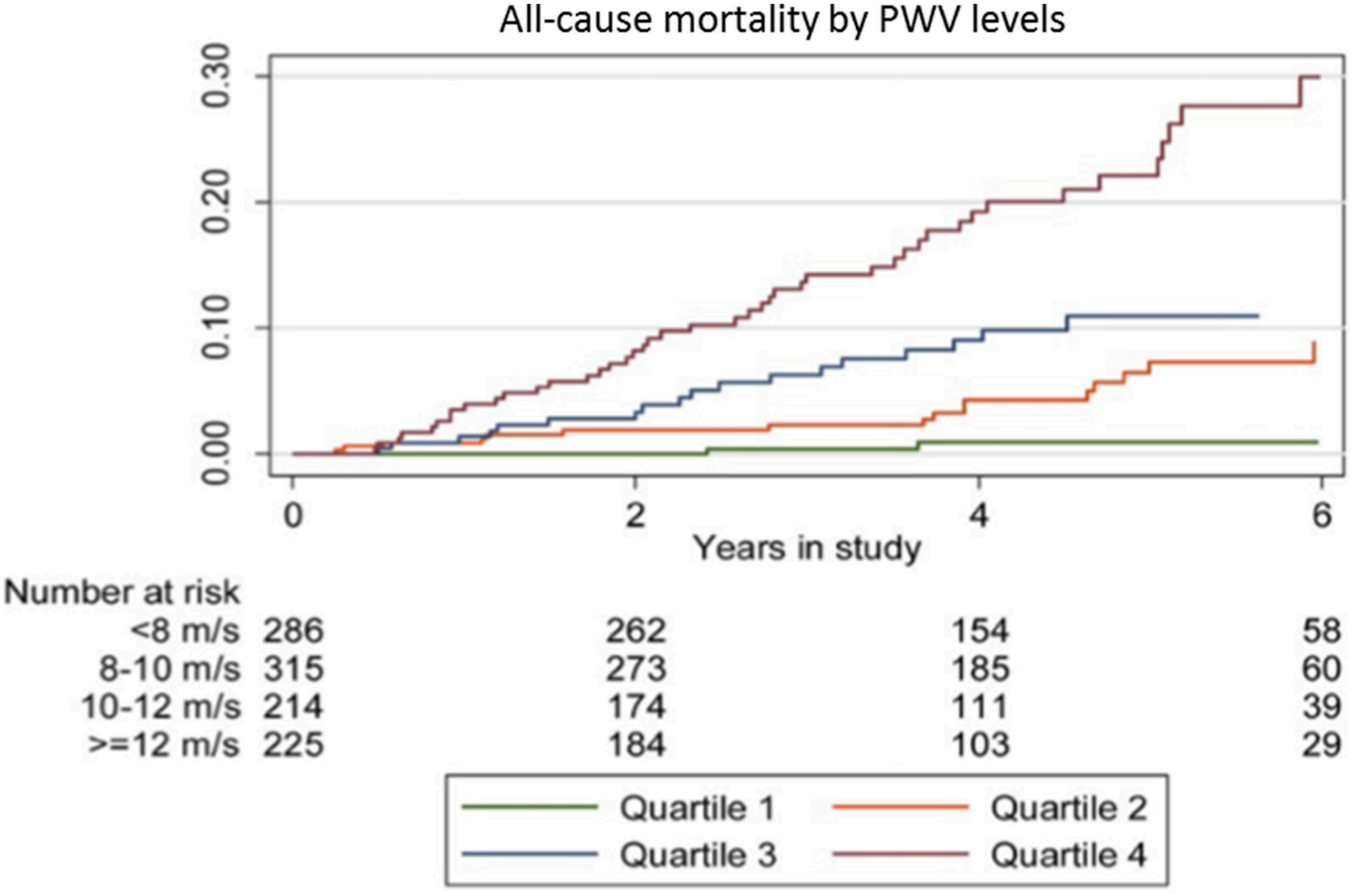
Kaplan-Meier plot of all-cause mortality according to PWV quartiles in a prospective study of 1,040 renal transplant recipients with a median follow-up of 4.2 years. Reprinted with permission from Dahle et al. (53).

Further to the above, the association between arterial stiffness and all-cause mortality has been explored in two recent retrospective studies. The first one, conducted by Dahle et al. (53) included 1,040 renal transplant recipients to whom carotid-femoral PWV was measured 8 weeks after kidney transplantation. Approximate PWV quartiles were defined by cut-offs at 8, 10, and 12 m/s. During a median follow-up of 4.2 years 82 patients died. The association of PWV and mortality showed a ceiling effect and PWV was truncated at 12 m/s. Each 1 m/s increase in PWV up to 12 m/s, was significantly associated with mortality (HR 1.36, 95% CI 1.14 to 1.62, *p* = 0.001) (Figure 2). An interquartile range increase of 3.8 m/s in PWV tripled the risk of mortality (HR 3.21, 95% CI 1.63 to 6.31), an effect similar to the effect of 1 interquartile increase in age (21.6 years, with an estimated HR of 3.06, 95% CI 1.87 to 5.29) (53). Finally, in the most recent study assessing the association between arterial stiffness, mortality and graft survival in renal transplant recipients, Cheddani et al. (54) included 220 patients that were evaluated for PWV 3 months after transplantation. Among those, 169 repeated the evaluation at 12 months. During a median follow-up of 5.5 years, death and graft loss occurred in 10 and 12 patients, respectively. c-f PWV 3 months after transplantation was an independent risk factor for mortality (HR: 1.38, 95% CI 1.18 to 1.62, *p* < 0.001). Mortality was also significantly associated with c-f PWV 12 months after transplantation (HR 1.34, 95% CI 1.1 to 1.64, *p* = 0.004), but not with aortic stiffness change between 3 and 12 months (HR 1.09, 95% CI 0.71 to 1.76, *p* = 0.696) (54).

#### Prospective Studies

Bahous et al. (55) prospectively evaluated large artery stiffness in 106 renal transplant recipients with a mean age of 43 ± 14 years, by determining non-invasively aortic PWV with the Complior device. During the follow-up period (mean duration 54.3 ± 28.9 months), the following parameters were studied: characteristics of the renal graft, degree of renal insufficiency, number of acute rejections, cardiovascular risk factors, drug medications and cardiovascular complications. The study revealed that carotid-femoral PWV was significantly higher in renal transplant recipients when compared to normotensive men and women of the same age (*p* < 0.0001 for both genders), despite a lower MBP compared to normal controls (87.8 ± 13.5 mm Hg vs. 94.5 ± 6.7 mm Hg for men, *p* < 0.0001 and 85.7 ± 12.8 mm Hg vs. 89.4 ± 7 mm Hg, respectively for women, *p* = 0.068). Moreover, independently of age and BP levels, PWV was influenced by two factors: presence of acute rejection (β 1.15, standard error of β 0.44, *p* = 0.01) and increased tobacco consumption (β 0.02, standard error of β 8.3, *p* = 0.025). These two factors, along with donor age also influenced the decrease in GFR observed after transplantation (β −15.87, standard error of β 3.73, *p* < 0.0001 and β −0.12, standard error of β 6.13, *p* = 0.058, respectively). Finally, the authors reported that the occurrence of renal and/or cardiovascular events following transplantation was influenced by two factors: heart rate (β, 7.16; *p* < 0.001) and PWV (β, 0.25; *p* < 0.006). When PP was multiplied by heart rate, this product was a significant (HR 3.7; *p* < 0.02) and independent factor influencing cardiovascular events in transplanted patients, in addition to a past history of cardiovascular events (HR 1.16; *p* < 0.04). The study is limited, however, by the lack of details on the methodology of the analysis (55).

Claes et al. (56) conducted a prospective study in order to investigate the prognostic value of arterial stiffness and aortic calcifications in 253 renal transplant recipients. Carotid-femoral PWV was assessed in a subgroup of 115 patients and aortic calcifications (AC) was assessed by means of lumbar X-ray. AC were present in 61% of patients. The primary endpoint for this study were cardiovascular events. After a mean follow-up of 36 months, 32 CV events occurred in the overall group and 13 events in the PWV subgroup. When accounting for age, gender, and cardiovascular history, aortic calcification score (HR, 1.09 per 1 unit increase; 95% CI 1.02 to 1.17) and PWV (HR 1.45 per 1 m/s; 95% CI 1.16 to 1.8) remained independent predictors of cardiovascular events in Cox-regression analyses. Using ROC-analysis, the area under the curve for the prediction of CV events was 0.80 and 0.72 for sum aortic calcification and PWV, respectively. This study indicated that both arterial stiffness and aortic calcifications are strong and independent predictors of future cardiovascular events in non-selected renal transplant recipients and should be used in risk stratification (56). In a recent single-center observational prospective study (57), 37 kidney transplant recipients with no history of vascular event were evaluated in terms of vascular calcification. Among others, carotid-femoral PWV (cfPWV) and carotid-radial PWV (crPWV) were measured using applanation tonometry before and 1 year after transplantation. The study showed that pre-transplant CRP level (HR 1.660, *p* = 0.007) and PWV ratio (cfPWV/crPWV) (HR 7.549, *p* = 0.045) predicted cardiovascular events (57).

Bahous et al. (58) conducted a study that included 95 recipients of living donor kidneys and their corresponding donors, aiming at determining the contribution of donor characteristic especially large artery stiffness, in addition to recipient parameters, to late post-transplant cardiovascular and renal graft outcome. The study revealed a borderline significant association of donor aortic PWV with the composite outcome (occurrence of a fatal or nonfatal cardiovascular event and/or doubling of serum creatinine or development of ESRD) (RR 1.8, 95% CI 1 to 3.4, *p* = 0.05) in renal transplant recipients. When renal and cardiovascular outcomes were separately analyzed, recipient eGFR and donor PWV were significant determinants of the renal outcome (HR 0.26, 95% CI 0.14 to 0.4, *p* < 0.0001 and HR 1.9, 95% CI 1.2 to 3.0, *p* = 0.02, respectively) and previous history of cardiovascular events was the only significant determinant of the cardiovascular outcome (HR 3.5, 95% CI 2.1 to 8, *p* = 0.001) (58).

## Studies Evaluating Arterial Stiffness Before and After Renal Transplantation

Prospective observational studies evaluating the impact of successful transplantation on arterial stiffness of the recipients are presented in Table 3. Most of them are based on measurements before and some when after surgery. In the first study to explore the long-term effects of renal transplantation and hemodialysis on arterial stiffness, Keven et al. (59) prospectively assessed c-f PWV using SphygmoCor device in 28 renal transplanted patients before and 12 months after renal transplantation and in 23 patients on hemodialysis at baseline and 12 months later. In renal transplant recipients PWV significantly decreased from 7.8 ± 1.8 to 6.2 ± 1.6 1 year after transplantation, which was a significant reduction compared to hemodialysis patients (*p* < 0.0001) (59). In a subsequent study, Ignace et al. (60) measured c-f PWV and heart-rate adjusted AIx (AIc75) before and 3 months after transplantation in 52 renal transplant recipients, using the Complior device. After adjusting for the reduction in mean BP, c-f PWV decreased significantly from 12.1 ± 3.3 to 11.6 ± 2.3 m/s (*p* < 0.05). Moreover, in an analysis stratified by age, this improvement was only present in patients older than 50 years of age as compared with patients younger than 50 years of age (-5.5 ± 2.2 vs. 2.1 ± 1.9 %, *p* < 0.05). As far as AIx75 is concerned, it decreased from 22 ± 11 to 14 ± 13% (*p* < 0.01), but this reduction was not age-dependent (60).

**Table 3:** Studies evaluating arterial stiffness before and after renal transplantation or in different time-points after renal transplantation.

**Study ID**	***N***	**Population characteristics**	**Time points**	**Arterial stiffness assessment**	**Result**
**STUDIES ASSESSING PWV BEFORE AND AFTER RENAL TRANSPLANTATION**					
Keven et al. (59)	28	Renal transplant recipients	Before and 1-yr after RT	c-f PWV *SphygmoCor*	PWV (m/s) from 7.76 ± 1.8 to 6.16 ± 1.6 1 year after RT (*p* < 0.0001)
Ignace et al. (60)	52	Renal transplant recipients	Prior to and 3 months after RT	c-f PWV, augmentation index (AIx) *Complior*	c-f PWV from 12.1 ± 3.3 to 11.6 ± 2.3 m/s (*p* < 0.05) Patients older than 50 vs. patients younger than 50 change-5.5 ± 2.2 vs. 2.1 ± 1.9%, (*p* < 0.05) AIx from 22 ± 11 to 14 ± 13% (*p* < 0.01), not age-dependent
Hornum et al. (61)	40	Renal transplant recipients	Before RT and after 12 months	c-f PWV AIX *SphygmoCor*	AIX from 27% (17,–33) to 14% (7,–25) (*P* = 0.01) after 1 year c–f PWV did not change significantly after RT
Hotta et al. (62)	58	Renal transplant recipients	Preoperatively and 6 months postoperatively	baPWV *volume-plethysmographic apparatus*	PWV (m/s) from 15.9 ± 4.5 to 14.3 ± 2.6 6 months later (*p* < 0.01)
Kovacs et al. (63)	17	Renal transplant recipients	Before RT and 24 h, and 1 and 2 weeks after surgery	PWV, AIx, PP, systolic area index, diastolic area index, diastolic reflection area *Tensiomed Arteriograph*	PWV (m/s) from 13.36 ± 3.07 m/s to 9.56 ± 3.47 m/s day 1, 11.19 ± 3.5 m/s day 7 to 8.25 ± 1.93 m/s day 17 (*p* = 0.0075) AIx (%) from 41.97 ± 11.88 m/s to 26.09 ± 10.92 m/s day 1, 23.02 ± 16.5 m/s day 7, 21.96 ± 11.98 m/s day 17 (*p* = 0.013)
M. Kaur et al. (64)	23	Renal transplant recipients	Before and at 3 and 6 months after RT	c–f PWV, augmentation index (AI) and central pulse pressure (PP) *Sphygmocor*	c–f PWV from 8.65 ± 2.02 m/s before to 8.62 ± 3.23 m/s (NS) at 3 months and to 8.06 ± 2.54 m/s (NS) at 6 months AI (%) from 27.7 ± 11.3 before to 17.1 ± 9.0 (*P* < 0.05) at 3 months and 13.8 ± 12.4 (*P* < 0.05) at 6 months PP (mmHg) from 41.7 ± 13.9 before to 33.0 ± 11.1 (*P* < 0.05) at 3 months and 30.1 ± 9.8 (*P* < 0.05) at 6 months
Ro et al. (65)	67	Renal transplant recipients	Before surgery and 6 months, 1 and 2 years after	BaPWV *Volume—plethysmographic apparatus*	baPWV prior to RT and 6 months, 1 year, and 2 years after RT 1533 ± 261 cm/s, 1417 ± 254 cm/s, 1414 ± 285 cm/s, and 1384 ± 233 cm/s, respectively baPWV improved at 6 months (*P* < 0.05), but there were no changes between 6 months and 2 years after RT
Kim et al. (52)	171	171 ESRD pts eligible for Tx, in 84 of whom follow-up baPWV was available	Before Tx and 1 year after Tx	baPWV *VP-1000 BP203RPEII (Colin Company, Kyoto, Japan)*	The post-transplant baPWV was significantly decreased compared to that of pre-transplant rates (14.18 ± 2.35 vs. 15.17 ± 2.93 m/s, *p* < 0.05), and 86.9% patients showed no progression of arterial stiffness
Bilal Aoun et al. (66)	15	Children renal transplant recipients	Every 6 months before RT and 6 months after	c-f PWV AI *SphygmoCor*	PWV before was 6.1 ± 1.3 m/s and 6.5 ± 1.4 m/s post-RT (*P* = 0.46) AI pre-RT was 6.93 ± 11.41% vs. −0.53 ± 17.05% post-RT (*P* = 0.15)
**STUDIES ASSESSING PWV ONLY AFTER RENAL TRANSPLANTATION**					
Delahousse et al. (67)	74	Cadaveric renal transplant recipients	3 and 12 months after transplantation	c-f PWV *Complior*	MBP-adjusted PWV decreased 0.43 m/s between 3 and 12 months in recipients of young-donor (17–41yr) kidneys (*P* = 0.028) PWV increased in recipients of old-donor (53–70 yr.) kidneys from 9.97 ± 1.70 to 10.25 ± 1.98 (*P* = 0.022)
Birdwell et al. (68)	66	Renal transplant recipients	within one month of transplant (baseline) and 12 months post	c-f PWV *SphygmoCor*	Median PWV score was 9.25 vs. 8.97 m/s at baseline vs. month 12 (median change of −0.07, *p* = 0.7)
Karras et al. (69)	161	Renal transplant recipients	3 and 12 months after transplantation	c-f PWV *SphygmoCor*	PWV from 10.8 m/s (10.5–11.2 m/s) (at month 3) to 10.1 m/s (9.8–10.5 m/s) (at month 12) (*P* < 0.001) PWV reduction larger in the living donor allograft KT (*P* < 0.001) Extended criteria donors' group +0.1 (−0.4 to 0.4) Standard criteria donors' group −0.7 (−1 to −0.4) (*P* < 0.01)
Saran et al. (70)	181	Renal transplant recipients	Early postoperative period (2–7 postoperative days) Late period (6 months to 27 years) after (RT)	c-f PWV *Schiller BR-102 plus PWA device*	Average PWV in the early period after RT was 8.02 ± 2.21 m/s and in the late period 8.09 ± 1.68 m/s (*P* = 0.777)

Hornum et al. (61) measured c-f PWV and AIx using Sphygmocor before transplantation and 12 months after it in 40 renal transplant recipients; c–f PWV did not change significantly 1 year after transplantation, although AIx was reduced from 27% (17–33) to 14% (7–25) (*p* = 0.01) (61). Another study that explored the possible amelioration of arterial stiffness in ESRD patients undergo successful kidney transplantation (62) brachial-ankle PWV was measured preoperatively and 6 months postoperatively in 58 renal transplant recipients with a plethysmographic method. A significant decrease of baPWV from 15.9 ± 4.5 to 14.3 ± 2.6 m/s (*p* < 0.01) at 6 months post-transplantation was observed (62). In another short-term prospective longitudinal study (63), arterial stiffness parameters measured in 17 primary transplant patients before surgery, and 24-h, 1 and 2.5 weeks after it. The study showed a significant decrease in PWV from 13.36 ± 3.07 m/s to 9.56 ± 3.47 m/s the first postoperative day, to 11.19 ± 3.5 m/s at postoperative day 7 and 8.25 ± 1.93 m/s at postoperative day 17 (*p* = 0.0075). Respectively, AIx decreased from 41.97 ± 11.88 to 26.09 ± 10.92% the first postoperative day, to 23.02 ± 16.5% at postoperative day 7 and to 21.96 ± 11.98% at study end (*p* = 0.013) (63).

In another relevant study, Kaur et al. (64) measured c-f PWV, AIx and central pulse pressure using Sphygmocor in 23 renal transplant recipients before and 3 and 6 months after transplantation. PWV decreased insignificantly from 8.65 ± 2.02 m/s before to 8.62 ± 3.23 m/s at 3 months and to 8.06 ± 2.54 m/s at 6 months, while AIx (%) decreased significantly from 27.7 ± 11.3% before to 17.1 ± 9.0 % (*p* < 0.05) at 3 months and 13.8 ± 12.4 % (*p* < 0.05) at 6 months; a decrease in pulse pressure from 41.7 ± 13.9 before to 33.0 ± 11.1 (*p* < 0.05) at 3 months and to 30.1 ± 9.8 mm Hg (*p* < 0.05) at 6 months was also noted (64). In a more recent study, Ro et al (65) measured baPWV in 67 renal transplant recipients before surgery and 6 months, 1 and 2 years after it. baPWV prior to kidney transplantation and 6 months, 1 year, and 2 years after transplantation was 1,533 ± 261 cm/s, 1,417 ± 254 cm/s, 1,414 ± 285 cm/s, and 1,384 ± 233 cm/s, respectively. baPWV improved significantly at 6 months (*p* < 0.05), but there were no changes between 6 months and 2 years after transplantation (65). Finally, in the aforementioned study of Kim et al., in which baPWV was measured pre and 12 months post-transplant in 84 patients, the post-transplant baPWV was significantly decreased compared to that of pre-transplant levels (14.18 ± 2.35 vs. 15.17 ± 2.93 m/s, *p* < 0.05). Logistic regression analysis revealed that higher body mass index (OR 1.348, 95% CI: 1.049 to 1.732, *p* < 0.05) and the degree of increase in calcium levels (OR 4.255, 95% CI: 1.492 to 12.132, *p* < 0.05) were independent risk factors that affected baPWV after kidney transplantation (52). The above findings was not confirmed in children renal transplant recipients, according to a study (66) evaluating 15 children before and 6 months after transplantation. PWV was 6.1 ± 1.3 m/s vs. 6.5 ± 1.4 m/s before and post-transplantation, respectively (*p* = 0.46). AIx before transplantation was 6.93 ± 11.41% vs. −0.53 ± 17.05% post-transplantation (*p* = 0.15) (66).

## Studies Evaluating Arterial Stiffness in Different time Points After Renal Transplantation

In addition to the above, there are a few prospective observational studies that performed on measurements in different time points after renal transplantation. Delahousse et al. (67) used the Complior device to measure c-f PWV in 74 renal transplant recipients from deceased donors 3 and 12 months after transplantation. Mean BP-adjusted PWV decreased by 0.43 m/s between 3 and 12 months in recipients of young-donor (17–41 years of age) kidneys (*p* = 0.028), while it increased in recipients of old-donor (53–70 years of age) kidneys from 9.97 ± 1.70 to 10.25 ± 1.98 (*p* = 0.022) (67). Subsequently, Birdwell et al. (68) used Sphygmocor to assess c–f PWV within 1 month of transplant (baseline) and 12 months post-transplant in 66 renal transplant recipients Median PWV score was 9.25 vs. 8.97 m/s at baseline and month 12, respectively but the change was not significant (median change of −0.07, *p* = 0.7) (68).

In perhaps the most interesting of these studies, Karras et al. (69) evaluated arterial stiffness with c–f PWV in 161 renal transplant recipients, 3 and 12 months after transplantation. These recipients were separated in three different groups based on their donors, i.e., recipients from living donors, recipients from standard criteria donors, and recipients from extended criteria donors. Mean PWV decreased from 10.8 m/s (95% confidence interval, 10.5–11.2 m/s) at month 3 to 10.1 m/s (95% confidence interval, 9.8–10.5 m/s) at month 12 (*p* < 0.001). PWV reduction from month 3 to month 12 was significantly larger in patients with the living donor allograft compared to those with the deceased donor allograft (*p* < 0.001). When the extended criteria donor (ECD) group were compared to the standard criteria donor (SCD) group, the change in PWV also differed significantly, −0.7 (−1 to −0.4) in ECD vs. +0.1 (−0.4 to +0.4) in SCD (*p* < 0.01) (69). Finally, very recently, Saran et al. (70) measured c-f PWV using Schiller BR-102 plus PWA device in 181 renal transplant patients in two different postoperative periods. The early postoperative period was between 2 and 7 postoperative days and the late was 6 to 27 years after transplantation. In contrast to most of the above findings, the authors noted no significant difference between the average PWV in the early period after renal transplantation (8.02 ± 2.21 m/s) and in the late period (8.09 ± 1.68 m/s) (*p* = 0.777) (70).

## Conclusions

Kidney transplantation is the treatment of choice in patients suffering from ESRD, yet cardiovascular risk in renal transplant recipients remains significantly higher than that of the general population. This excess risk is not fully explained by the burden of traditional cardiovascular risk factors present in renal transplant recipients. As increased arterial stiffness is a prominent feature of vascular changes in patients with CKD and ESRD and has been repeatedly associated with increased risk of cardiovascular events and mortality in these conditions, different types of studies have been conducted in order to assess the degree of arterial stiffness in renal transplant recipients and its associations with other risk factors but also with future cardiovascular risk, graft survival and overall mortality.

Several cross-sectional studies discussed in detail herein, showed that higher PWV in renal transplant recipients was associated with various risk factors, co-morbidities, and associated target-organ damage including age, pre-transplant diabetes, increased ambulatory BP, increased waist circumference and visceral fat mass, smoking, coronary artery calcification, and left ventricular hypertrophy, but also with previous episodes of acute renal rejection, renal graft dysfunction and previous time on dialysis. Other studies have assessed the course of arterial stiffness before and after kidney transplantation. In most cases, arterial stiffness measured with PWV was markedly reduced after kidney transplantation. Of note, in some studies this improvement was shown to be age-dependent, suggesting an added cardiovascular risk reduction in older patients and was also more marked in cases of transplantation from living donors. In the subset of studies examining PWV in different timepoints after kidney transplantation (varying from 1 week to 2 years), mixed results were noted, with some studies showing that arterial stiffness was partly reversed during follow-up and that this improvement was dependent on donor age and greater in patients receiving a renal graft from a living donor, whereas other studies suggesting that arterial stiffness levels were similar in the early and late post-transplantation period Finally, and perhaps most importantly, in almost all studies evaluating the role of arterial stiffness as a predictor of future adverse outcomes, PWV was shown to be an independent predictor of cardiovascular events, loss of renal function and overall mortality. Thus, existing evidence suggests that increased arterial stiffness is a major pathophysiological player involved in the adverse cardiovascular profile of renal transplant recipients. As of this writing, however, there are not any clinical trials in transplant recipients (or any other population) aiming to assess whether therapeutic interventions to reduce of arterial stiffness would improve patient outcomes. To this scope, mechanistic studies to identify the major mechanisms through which renal transplantation beneficially affects PWV are also required. Overall, further studies are urgently warranted to better define the associations of PWV with other prominent risk factors, the change and evolution of arterial stiffness after kidney transplantation, its long-term prognostic significance and whether it could represent an additional therapeutic goal in order to improve patient and graft survival after renal transplantation.

## Author Contributions

MK and EX wrote the first draft of the manuscript. SM wrote sections of the manuscript and checked the Tables for intellectual content. PS and JB conceptualized the article, wrote sections of the manuscript, and checked the manuscript for intellectual content.

### Conflict of Interest Statement

The authors declare that the research was conducted in the absence of any commercial or financial relationships that could be construed as a potential conflict of interest.
